# Meeting report of the sixth annual tri-service microbiome consortium symposium

**DOI:** 10.1186/s40793-023-00523-8

**Published:** 2023-08-02

**Authors:** Ida G. Pantoja-Feliciano De Goodfellow, Richard Agans, Robyn Barbato, Sophie Colston, Michael S. Goodson, Rasha Hammamieh, Kristy Hentchel, Robert Jones, J. Philip Karl, Robert Kokoska, Dagmar H. Leary, Camilla Mauzy, Kenneth Racicot, Blake W. Stamps, Vanessa Varaljay, Jason W. Soares

**Affiliations:** 1 Soldier Effectiveness Directorate, United States Army Combat Capabilities Development Command Soldier Center, 10 General Greene Ave, Natick, MA 01760 USA; 2 711th Human Performance Wing, Air Force Research Laboratory, Wright-Patterson AFB, Dayton, OH USA; 3 United States Army ERDC Cold Regions Research and Engineering Laboratory, Hanover, New Hampshire USA; 4 United States Naval Research Laboratory, Washington D.C., USA; 5 Medical Readiness Systems Biology, Walter Reed Army Institute of Research, Silver Spring, MD USA; 6 Office of Naval Research, Arlington, VA USA; 7 Military Nutrition Division, United States Army Research Institute of Environmental Medicine, Natick, MA USA; 8 Physical Sciences Directorate, United States Army Research Laboratory, United States Army Research Office, Research Triangle Park, Durham, NC USA; 9 Materials and Manufacturing Directorate, Air Force Research Laboratory, Wright-Patterson AFB, Dayton, OH USA

**Keywords:** Human microbiome, Environmental Microbiome, Mycobiome, Stress response, Earth and Space, Biotechnology, Microbiome Engineering, Military Microbiome Analysis, Gut Microbiome

## Abstract

The Tri-Service Microbiome Consortium (TSMC) was founded to enhance collaboration, coordination, and communication of microbiome research among DoD organizations and to facilitate resource, material and information sharing amongst consortium members, which includes collaborators in academia and industry. The 6th Annual TSMC Symposium was a hybrid meeting held in Fairlee, Vermont on 27–28 September 2022 with presentations and discussions centered on microbiome-related topics within seven broad thematic areas: (1) Human Microbiomes: Stress Response; (2) Microbiome Analysis & Surveillance; (3) Human Microbiomes Enablers & Engineering; (4) Human Microbiomes: Countermeasures; (5) Human Microbiomes Discovery - Earth & Space; (6) Environmental Micro & Myco-biome; and (7) Environmental Microbiome Analysis & Engineering. Collectively, the symposium provided an update on the scope of current DoD microbiome research efforts, highlighted innovative research being done in academia and industry that can be leveraged by the DoD, and fostered collaborative opportunities. This report summarizes the activities and outcomes from the 6th annual TSMC symposium.

**Supplementary Information:**

The online version contains supplementary material available at 10.1186/s40793-023-00523-8.

## Introduction

The Tri-Service Microbiome Consortium (TSMC) was chartered in 2016 to enhance collaboration, coordination, and communication of microbiome research among DoD organizations and to facilitate resource, material and information sharing among consortium members. Towards those goals, and to discuss applications and implications of DoD-associated microbiome research, the TSMC hosts an annual symposium that includes subject matter experts from federal/state agencies, DoD-affiliates, academic institutions, and industry [1–5]. The 2022 annual symposium was a hybrid meeting with the in-person option held in Fairlee, Vermont on 27–28 September 2022 concurrent with the virtual attendance, with presentations and discussions centered on microbiome-related topics within seven broad thematic areas: (1) Human Microbiomes: Stress Response; (2) Microbiome Analysis & Surveillance; (3) Human Microbiomes Enablers & Engineering; (4) Human Microbiomes: Countermeasures; (5) Human Microbiomes Discovery - Earth & Space; (6) Environmental Micro & Myco-biome; and (7) Environmental Microbiome Analysis & Engineering (Table S1; https://www.ues.com/hubfs/TSMC_Annual_Program_2022.pdf). 43% of the speakers were female and 15% of the speakers were from underrepresented minorities. Twenty-nine speakers were from DoD laboratories, seven were from non-DoD government laboratories, eight were from academic institutions (two from the Uniformed Services University, two from the United States Air Force Academy, one from the United States Naval Academy, one from the United States Military Academy, and two from MIT, and two were from non-government industry partners, including a non-profit (Table S1). In lieu of a poster session, three sessions of 10 min ‘lightning talks’ were included in the agenda followed by a question-and-answer session paneled by the lightning talk presenters. This report details the symposium activities to facilitate communication of current research efforts and foster potential collaboration across scientific communities.

## Opening remarks

The TSMC annual symposium commenced with an overview of the TSMC given by TSMC Chair Mr. Jason Soares (DEVCOM SC), and Vice-Chair Dr. Michael Goodson (AFRL), highlighting the breadth of the research areas covered by consortium members and the overall objectives of the organization, including monitoring and reporting global advances in microbiome research. They provided a summary of previous TSMC-led meetings to date as well as the structure of the consortium, which stands at more than 275 members, and its position within the wider DoD Biotechnology Community of Interest (CoI). The DoD Biotechnology CoI was established by the Office of the Under Secretary of Defense for Research and Engineering to coordinate, integrate, and synchronize the DoD’s biotechnology research and continually assess new developments in the field [6]. Following the introduction, a discussion commenced regarding the technology vision for an era of competition and the importance of the biotechnology focus, foundation building, and succeeding through teamwork. The President’s Executive Order on Advancing Biotechnology and Biomanufacturing Innovation for a Sustainable, Safe, and Secure American Bioeconomy [7] was referenced, as was biotechnology’s role in furthering societal goals, the need to promote standards in the biological data ecosystem, and the DoD Biotechnology Modernization Framework, which includes the collaboration of academia and small business, centered on creating an industrial biotechnology base in the US.

In a special session called ‘User Engagement’, the audience had the opportunity to hear directly from military representatives about their experiences during their deployment. In this informal but insightful discussion, experiences recruiting volunteers for microbiome studies were discussed, specifically collecting samples during a submarine deployment. Having leadership involved in the process is essential, especially if sample collection could be added to the plan of the day. Also, recruiting study participants was more likely if the study goals and purpose were explained to them, and flexibility in sampling was essential to accommodate the duties of the participants. The second discussion was towards experiences of deployment and some of the operational stressors encountered, including how operational tempo varied during and between deployments. The importance of acclimating to the specific cultures and to be respectful in that environment was highlighted, even if that may present situations that are counter to usual hygiene practices. The two topics clearly engaged the in-person and virtual audience in an interactive discussion about the experiences that duty personnel and medics face during their active duties.

## Human microbiome: stress response

After the exceptional introduction to this year’s meeting, the first session of the day started with four prominent topics in the area of Human Microbiome: Stress Response. Each body site has a specific microbiome composition and function that responds to stressors from the environment. The impact of submarine deployment on mood, dietary practices, and gut microbiota was discussed. The unique population and occupational conditions experienced by submariners, such as limited dietary options, increased occupational and social stressors, circadian rhythm disruption, limited space for exercise, and reduced Vitamin D, affected gut microbial composition during deployment, with self-reported fatigue exhibiting significant correlation with within-subjects gut microbiome diversity changes.

Bioenergy homeostasis regulation by microbiota was also highlighted in this session. Using mouse models [8], host aging affected microbial energy outputs, enrichment of anaerobic metabolites and alternate energy cycles, while younger cohorts maintained a greater aerobic function. Microbial pathways related to bioenergy and immune function were also affected by radiation exposure of the host, leading to significant and unique alterations in metagenome network activation with paired decreases in metabolite networks.

The nasal microbiome evolution and the influence of military training on SSTIs [9] was also highlighted. The estimated cost of SSTIs is around $14–32 million per year, encompassing 80,000 yearly medical visits and 1300 hospital admissions. The correlation between nasal *Staphylococcus aureus* colonization in patients with SSTIs was described, with roughly 40–60% of patients testing positive for *S. aureus* in nasal samples, and 80% incidence of the SSTI-causing organism matching the nasal-colonizing strain. The importance of subject metadata, multi-body site sampling, and potential for probiotic therapeutics was emphasized, and is part of ongoing efforts.

The session closed with a discussion of discerning alterations in gut, oral, and skin microbiomes associated with chronic physical and psychological health conditions and medication use, as part of the United States-Veteran Microbiome Project [10–12]. The goal is to better understand the relationship between human microbiota, health conditions in veterans, and effectiveness of medications. There are unique symptoms present in veteran populations, particularly advanced and rapid aging. Multiple classes of medications, including cardiovascular and psychiatric medications, were correlated to significant microbial community shifts. There is a need for more research on the interplay between microbiome, medication, and health outcomes, as well as the importance of including medication use as a variable, longitudinal studies, and partnerships between clinical and research initiatives.

## Microbiome analysis & surveillance

The ‘Microbiome Analysis & Surveillance’ session started with a discussion on fecal microbiome composition and its changes upon non-lethal radiation exposure. Long-term effects of low dose radiation on microbiomes of female and male rats showed differences in diversity among the genders based on 16 S sequencing results.

Microbiome surveillance is also included in the digital engineering for chemical, biological, and radiation (CBR) defense called the Navy Shipboard CBRN Performance Model that is currently ongoing. This model incorporates the human microbiome changes and data from vital sign devices to simulate performance. Similarly, the use of next-generation sequencing and metagenomics for aerobiological surveillance was highlighted. Researchers have found that sequencing was feasible and corresponded to microscopy findings of pollen allergens and further identified environmental bacteria and fungi. Controlled laboratory studies to address sensitivity, specificity, and speed needed to develop field-deployable air quality surveillance system are ongoing.

Finally, a promising radiation countermeasure involving oral suspension of synthetic genistein nanoparticles, called BIO300 [13] was highlighted. Mice treated by BIO300 recovered from radiation damage to their gut microbiome within 14 days with re-occurrence of commensal bacteria, which were lost upon radiation exposure. An inflammatory metabolic phenotype was observed immediately upon radiation exposure; however, BIO300-treated mice showed corrective inflammatory response that correlated with a restored microbiome.

## Human microbiomes: countermeasures

Respiratory and gastrointestinal tract infections can impact force readiness. As part of the next session ‘Human Microbiomes: Countermeasures’, a systematic review and meta-analysis [14] about orally ingested probiotics [15], prebiotics [16], and synbiotics as countermeasures for RTI and GTI [17, 18], was discussed. Their main observations showed that (1) probiotics reduce risk of RTIs by 9%, reduced the duration of the RTI by 1 day, and also reduced its severity, (2) fermented dairy gave the strongest response with no clear evidence for or against any one probiotic strain, (3) beneficial effects were not observed in physically active populations, (4) there was a trend of prebiotics, probiotics, and synbiotics reducing the risk of GTI, but it was not significant and (5) there was no effect on duration of GTI or total days, but it did reduce the overall risk by 21%. While there is promise for prebiotics, probiotics and synbiotics as prophylactics for RTI and GTI, there are currently not enough studies to recommend use in military populations.

A probiotic evaluation of the gut-muscle axis using an in vitro model system was evaluated. Interest in the gut-muscle axis has grown after studies have shown adding gut bacteria to germ-free mice caused improvements in muscle fiber, muscle weight%, and grip strength [19]. Musculoskeletal injuries in the military are prevalent in military environments, and probiotic interventions to reduce injury and/or speed recovery is an active area of research.

Continuing the discussion of probiotic intervention studies, in vitro models and analyses were highlighted. *Lactobacillus rhamnosus GG (LGG)* was isolated in 1985 by Gorbach and Goldin [20]. Some of the main characteristics of this microbe are that it survives through to the colon, can colonize human epithelial cells, can exert antimicrobial effects, has rapid growth, and has beneficial effects to human health. Researchers utilized the SHIME system to determine the effect of adding LGG to an existing microbial community. LGG was found to persists in the SHIME, as supported by qPCR and metagenomic sequencing data. 16S rRNA showed conserved phylum level trends across all donors without any differences over time. The largest difference in microbial communities were by colonic region, and no community structural shifts were observed after addition of LGG. However, profiles from untargeted metabolites show donor/site patterns and some separation by LGG addition. These data correlated with in vivo studies, suggesting that in vitro systems can contribute to teasing apart the processes and interactions that occur, hidden from our view, in vivo.

Additionally, a DoD-unique in vitro model system called the joint Army Automated Colon on a Bench (GI-jA^2^COB) was discussed, which is built on a commercial platform, including a small bowel model with resident microbiota, and a multistage large intestine model seeded with human fecal microbiota. Overall, the model represents a physiologically-relevant system that reaches a validated equilibrium after 2 weeks and thus ready for one week intervention challenges to understand impact of complex dietary inputs on microbial composition and functional capacity while also assessing the persistent or transient nature of the interventions on microbial community dynamics during the recovery phase. The in vitro system provides an ideal complement to animal and human studies.

## Environmental micro- & myco-biomes

Microorganisms are ubiquitous throughout the biosphere, occupying diverse niches including various extreme habitats. Environmental microbes collectively represent a wide breadth of biodiversity and are capable of a vast multitude of metabolic processes, which are important for their survival in their associated habitats. Understanding these physiological activities and how microbes respond to cues and perturbations in their environment in turn can provide insight into processes important to DoD operations such as biodegradation/bioremediation, biogeochemical cycling, biofouling, pathogen transmission, antimicrobial evolution/dissemination, and others. The work presented in this session proffer insights into some of these processes and the associated environmental microbiomes and/or mycobiomes.

Ongoing work exploring the electrical properties of melanin in its native state, namely in eumelanin fungal structures, was conferred as part of this session [21]. Signal propagation of sinusoidal and audio waveforms were measured across cultures of the melanized fungus, *Curvularia lunata*. Signal propagation in the range of 1 – 80 Hz was generally found to have minimal loss in power while signals in the range of 80 kHz – 20 MHz experienced rapid decay and attenuation. An audio waveform was successfully propagated albeit reduced in signal power. Future studies will focus on impedance at higher frequencies and relating melanin conductivity phenomena to electric circuit models.

Biocorrosion and the effect of microbiomes that can be found on and in aircraft systems was also discussed. Corrosion of aircraft, ships and other equipment leads to asset degradation, reduced availability and performance and billions of dollars in costs to the DoD [22]. Characterizing the microbial communities in contaminated areas can help to identify potential degraders and guide the development of methods to mitigate the adverse effects of microbiologically influenced corrosion. Results of a study that assessed the microbial communities on 29 locations on aircraft pre- and post-decontamination using JBADS were presented, a system that was developed for biothreat decontamination. Levels of potentially corrosion-causing microorganisms were found to be reduced following JBADS decontamination, suggesting that the system can be useful in the remediation of aircraft with deleterious microbial contamination.

Continuing the topic of biodegradation, the total (fungal and bacterial) microbiomes of surface-contaminated samples including four military aircraft and three vehicles was also presented. Since polyurethane (PU) coatings are used extensively on assets within the DoD as a protective layer, the meta-omics data were mined for enzymes that are associated with PU degradation. One finding suggested that fungal cutinase enzymes expressed by *Rachicladosporium spp.* were predominant in the aircraft interior locations and not highly expressed in samples taken from locations exposed to sunlight. Thus, an abiotic factor (sunlight) is thought to influence biodegradative processes. The data will be used to characterize both biological and correlated environmental factors, with the aim to develop practices to mitigate and/or prevent biodegradation.

And finally, characterizing the summer and winter planktonic microbiomes of the Chesapeake Bay, the largest estuary in the US, was a topic of interest and discussion. Water samples were collected at various locations in the vicinity of the Severn River, at different depths and seasonal timepoints and subjected to cultivation methods as well as targeted sequencing of the 16S rRNA gene and shotgun metagenomics sequencing to determine community/genetic composition. Results indicated a high similarity in surface waters at the different sampled locations with differences in taxonomic distribution by depth and season. There was also greater than expected variation in cultured species. Future work will focus on establishing the core microbiome of the estuary and include more rivers and locations in order to determine the stability of this microbiome over time, as well as any effects of anthropogenic and/or climatic forces that can be correlated to changes in the community structure.

## Lightning talks Session 1- human microbiome enablers & engineering

Opening this session, a group of researchers introduced a project to engineer a microbe to mitigate human performance decrements caused by periods of suboptimal sleep. Creatine has been shown to improve cognitive performance in sleep deprived individuals by increasing energy availability in the brain [23]. However, the half-life of creatine in the body is relatively short when supplemented orally. To overcome this issue, a research team engineered *Escherichia coli* Nissle 1917 to overexpress a gene that produces guanidinoacetic acid (GAA). GAA is converted to creatine in the liver. In vitro modeling demonstrated that the engineered probiotic functioned as expected even in a mixed community, and that the GAA produced was crossing the gut epithelium into the blood. Future studies are planned to take this engineered probiotic to human trials.

The DARPA’s Re-Vector Program was briefly discussed, with the goal of developing techniques to modify the skin organisms and their metabolic processes in order to diminish the impact of mosquito exposure. Mosquitoes are vectors that transmit several diseases in warfighters and civilians as well, like malaria, dengue fever, leishmaniasis, amongst others. There are some approaches that aim to decrease the exposure, including topical repellents, repellant-treated uniforms, bed net (treated or untreated) and chemoprophylaxis (when available). Although they are accessible, they have some logistical and safety challenges that have to be considered. This program is seeking to understand the volatilome to be able to engineer human skin microbe communities to alter key volatiles produced by humans and microbes and reduce attraction by testing 10 different microbial communities with reduced dehydrogenase lactate (mosquito attract). Also, the program will identify genes and pathways for the synthesis of repellents.

The microbiome community is aiming to give effective solutions to skin wounds specifically caused by the microorganism *Pseudomonas aeruginosa*. The goal is engineering a skin microbe to prevent infections caused by *P. aeruginosa* and try to find an antibiotic alternative to combat the infection. The 3 main aims of the project are: (1) produce a vector for *E.coli* product of a nanobody that targets *P. aeruginosa* surface proteins; (2) demonstrate *E.coli* nanobody excretion and attachment to *P. aeruginosa* surface proteins; and (3) evaluate nanobody capability to inhibit *P. aeruginosa*.

A challenge that researchers could face when conducting biological collections for microbiome studies is trying to discern the consequences of subjects self-sampling of fecal at home and shipping them to the research facility. This practice could be convenient for volunteers participating in a study but could cause several changes on the microbiota structure due to inconsistencies in temperature during the samples return, like the blooming of *Gammaproteobacteria*, results specifically found by the American Gut Project [24]. The idea is to develop better protocol designs including stabilization methods to avoid this type of variability that can alter final results.

The effects of consuming insoluble fibers (rice bran) on the gut microbial diversity relative to the consumption of soluble fibers was also a point of discussion. For this, researchers use the SHIME in vitro gut model system adding insoluble fiber, soluble fiber, and the control, using the gut microbiota of 3 different donors. It was observed that alpha diversity showed distinct changes in community and weighted unifrac analysis distributions were due to starting inoculum. Also, it was determined that the short chain fatty acids abundance changes were donor-dependent and increases in acetic acid, butanoic acid and propanoic acid were found in the distal community. The main results showed that insoluble fiber caused more changes on the gut microbiota than the soluble fiber.

Another important topic was the introduction of a platform that incorporates human immune cell types and the microbiome, with efforts on two main tissues, the ileum and skin. The goal is to reproduce key aspects of the human intestinal immune response with a focus on dendritic cells and responding T-cells that support the tridimensional and dimensional functionality of the model, to provide a host:microbiome interface that animal models can simulate. The idea of this model is to simulate the lamina propria with dentritic and T-cells, add an epithelia monolayer and introduce specific bacteria to evaluate the immune response.

Following this platform, a skin microbiome in vitro model was presented. This model incorporates microorganisms commonly found in skin microbiome representing it with less complexity (5 candidates at the moment) to test antimicrobials textiles, particularly important in military settings as soldiers may have limited access to shower/laundry for several days. The model should maintain tissue viability for 21 days to mimic extended textile wear period. These organisms are added to a solid substrate platform that simulates the skin and are exposed to a textile. The abundance of the microorganisms is measured by qPCR technique. As preliminary results, researchers found that one of the bacteria, *Micrococcus luteus*, is toxic to the tissue and TNF-alpha production occurred immediately after *M. luteus* introduction, due to stress. The team is still working on optimizing this model and the goal is to be able to co-culture aerobic and anaerobic microbes. The finality of this model is to be able to detect all the responses of the added textile to the microbial community.

Another approach presented was the implementation of an in vitro fermentation system that simulates the human colon conditions to evaluate the effects of probiotics in the generation of postbiotic fermentates and the possible role protecting from the damage of acute muscle inflammation. As part of the main results, the team found that the probiotic *Bacillus subtilis* produced mostly aromatic compounds and lipids; *Lactobacillus plantarum TWK10* mainly produced an increased profile of lipids, indole and flavonoid compounds; *Bifidobacterium bifidum* profiled anti-inflammatory compounds and *Bifidobacterium breve* presented large changes in indole profile and anti-inflammatory compounds.

Having the support of companies to promote scientific communication between different parties is crucial. With that perspective, BioMADE, the Bioindustrial Manufacturing Innovation Institute, was established in 2021 by the DoD. It is an independent non-profit for new biotechnology products with the goal of building a sustainable, domestic end to end bioindustrial manufacturing ecosystem looking for social responsibility, safety, sustainability, security, and education and workforce development. In addition to these points, BioMADE has additional focus areas: building awareness of bioindustrial manufacturing careers, preparing the future workforce with innovative education, and supporting the growth of the current workforce with world-class professional development.

## Lightning talks session 2: human microbiome discovery - earth & space

Human microbiome studies should take into account every aspect in the design and execution of the experiments that could affect the results, such as whether sequencing centers and platforms have an impact on microbiome results. There are several factors that could impact microbiome results like the sampling method, shipment, sample storage, DNA extraction method, primer selection, sequencing, and bioinformatics platforms. The primary objective was to compare four 16S sequencing centers results. The only difference between centers was in library normalization. Large differences in beta diversity were found between the two sites in the study, in terms of taxonomy. Researchers observed significant different communities between the two sequencing centers when comparing oral, skin, and fecal sequencing results between the two centers. It was proposed that a possible solution to this issue could be to sequence at the same facility, use positive controls (known abundance and include the same sample) and negative controls, be careful in the use of differential abundance tests and be aware of the unknown impact of different chemistries associated with same extraction and processing.

Testosterone supplementation could have an impact on the fecal microbiome and metabolome during energy deficit. Several previous studies have found testosterone is correlated with the host’s microbiota [25–27]. Training leads to energy deficit from physical activity and diet, which disrupts the endocrine system resulting in reduced testosterone levels and changes in gut microbiome. A study hypothesized that testosterone supplementation could prevent exercise-induced gut microbiome alterations during periods of energy deficit. A total of 50 male study participants were enrolled and divided into groups: half were given a testosterone supplement, while the other half were given a placebo. Within these two groups, participants were either given a eucaloric diet or a diet that would induce an energy deficiency under a controlled exercise regime. Fecal samples were taken at three timepoints. Results indicated that testosterone concentrations were maintained in placebo and increased in the supplemented group. However, no changes in alpha and beta diversity were observed between any of the groups. Changes in metabolomes and metabolites did not differ by treatment but did differ by time. They concluded that testosterone has minimal impact on changes in gut microbiome structure function and metabolic activity during sustained exercise and diet induced energy deficit.

It is being proposed that intestinal acylcarnitines could be a biomarker of IBD due to their role in bacterial metabolism. Acylcarnitines are membrane permeable intermediate metabolites of fatty acids and are an alternative energy source when short chain fatty acids are limited. Acylcarnitines are elevated during diabetes, cancer, heart failure, and nonalcoholic fatty liver disease. Fecal acylcarnitines have recently been found to be positively correlated with dysbiosis and fecal calprotectin and may be delivered to the gut via biliary secretion or by dysbiosis causing an increased amount of host epithelial cells and blood to enter the lumen. Acylcarnitines can be consumed by gut microbiota in lumen, and fecal acylcarnitines are positively correlated with *Enterobacteriaceae* and negatively correlated with the phyla *Bacteroidetes* and *Firmicutes*. Very little is known about utilization of acylcarnitines in the gut, but many bacteria can use carnitine as a carbon/nitrogen source, osmo-protectant and some bacteria can use the acyl chain for energy production. In conclusion, acylcarnitines may be an underappreciated modality which host can use to influence gut microbiome, and fecal acylcarnitines are elevated in patients with dysbiotic IBD.

The effect of chlorinated drinking water on the gut microbiota is an emerging topic of interest. Since the 1900s water chlorination limited spread of infectious diseases in the US. Chlorine is disruptive to cellular structures, to membrane and proteins, ATP, and causes DNA damage. Chlorine treatment is effective, but not selective. Many previous studies show chlorinated water does not affect human health. There were limited studies of the effect of chlorinated water on microbiome. In the study, male and female mice were exposed to chlorine and non-chlorine water. As part of their results, they found that chlorinated water does not alter alpha diversity and does not affect community structure. Chlorinated water may impact some metabolic pathways and altered community genetic potential, specifically for vitamin B12.

The interest on the effects of spaceflight on the fecal microbiome is rapidly increasing. To explore these, researchers have used a RR4 model, 40 mice in space and 40 on ground with/without Segmental bone defect (SBD), with the objective of examining the distinct effects of space flight and bone defects upon the entire fecal microbiome. Changes in bacteria, archaea, eukaryote, and virus at the phylum level abundance were observed (between SHAM and SAL/ground and space). There were significant differences in alpha diversity for flight vs. ground and beta diversity was significant for viruses. As conclusion, viral domain of microbiome had significant changes in the percent abundance due to microgravity and surgery.

Also, spaceflight-induced stress negatively impacted bone healing and increased tubercular separation and trabecular connectivity. In microbiome and brain transcripts, separation was observed between flight and ground sham samples. Neural pathway network was enriched in brain tissue with changes in neuromodulators and neurotransmitters. Impaired healing of segmental bone defect in space was potentially coordinated with inhibited pain perception and nociception, in space. In addition, there was a significant upregulation of brain-derived neurotrophic factor (BNDF) and signs of gut leakage in ileum epithelium. As part of the conclusions, it was mentioned that space increased the risk of bone injury and spaceflight delayed bone healing. Differentially abundant microbiota at species level were identified (*Lactobacillus johnsonni*) and found to be susceptible to both space flight induced stress and SBD.

## Lightning talks session 3 - environmental microbiome analysis & engineering

This session encompassed lightning discussions on environmental analysis and engineering. One of the topics was the identification of the mycobiome from aircraft topcoat using a decommissioned UH-60 helicopter sampled for molds and several isolates such as *Cladosporium*. The goal was to use this pilot study to test methods and evaluate microbial degradation and resilient topcoats and effective cleansers for mitigation. Another topic was to assess microbial threats in thawing permafrost using metagenomics sequencing. While it is widely known that permafrost is rapidly changing [28], there is less understood about the potential for emerging novel pathogens from thawing permafrost. This study sequenced samples and measured respiration collected at various locations in the CRREL permafrost tunnel. They found that with permafrost thawing, the microbial community is coming to life and assembled genomes from metagenomics sequencing will uncover the genetic context of potential novel pathogenic gene co-occurrence. Following on the permafrost theme, comparisons of GHG emissions from Alaska and Abisko permafrost focusing mainly on Abisko, were conveyed. Researchers measured permafrost gas fluxes using a LICOR GHG analyzer field system and found that rates differed between regions with varying properties in elevation, wetness, temperature. These data will be used as new parameters in the DRTSPORE model [29].

A study was briefly presented in which researchers attempted to “smell” wet soil to try to keep robots from accidentally getting stuck in muddy soils. Interestingly, the results showed there was a strong signal for terpene alcohols, geosmin and 2-methylisobornel, in wet/muddy soil. Further research will yield important relationships for monitoring soil properties and land use. Next, microbial activity in dust contaminated Antarctic snow at airfields near McMurdo Station was described. Windblown particulates can halt operations, but it is not known from where the soil particulates are being deposited and their activities. It was explained that the Pegasus airfield particulates were carbon-rich composites with strong microbial activities and several *Psychrobacterspp.* were isolated from these particulates after thawing. This session continued with a topic about using the DRTSPORE model to assess microbial activity in Arctic soil. This study used lab respiration and enzyme activities studies and showed that warm/wet soil produced the most carbon dioxide and enzyme activities to feed back into the DRTSPORE model. The discussion continued with the characterization of several microorganisms for low-temperature synthetic biology applications such as cell factories [30] and bioremediation [31]. The study goal is to engineer permafrost isolates to improve calcium carbonate mineralization [32] at lower temperatures. To close this session, a project on bioengineering and optimization of biocementation for potential space applications to reduce cargo load and provide dust mitigation was described. Researchers presented on their on-going work to clone and express marine sponge silicateins in *E. coli* and leverage the biosilification process to stabilize Martian soil regolith to ultimately provide biocementation and dust mitigation on Mars.

## Conclusions

The 6th annual TSMC symposium was a compendium of diverse research and initiatives underscoring the continuing importance and relevance of microbiome research within the DoD. Studying and characterizing human microbiomes remains paramount to understanding Warfighter physiology, particularly when perturbations occur in those microbiomes due to operational, environmental, physical, and medical factors, which may ultimately affect Warfighter performance. While gut microbiome research dominated the human-centric presentations, the microbiomes across human domains including the intersections of these microbiomes with health and performance, were emerging areas of interest. It is clear that multiple measures are needed to characterize and understand microbiomes, and tools such as multi-omic assessment and the use of organ models is essential to elucidate mechanisms and/or key interactions between host-microbe or microbe-microbe underpinning host phenotypes. Together, these studies can lead to the development of strategies for modulation or intervention that can mitigate adverse outcomes due to stressors and boost Warfighter resilience. A significant portion of the symposium was also spent discussing the research on environmental microbiomes, a vital area of research that explores the profiles and trends of natural and built environments across the globe and within areas of interest for the DoD. These studies will add critical understanding on how environmental conditions could affect important microbiome ecological process and also how they can affect directly and/or indirectly human microbiomes and the ability of warfighters and their materiel to perform in different environments. Moreover, since common goals and challenges exist for human and environmental microbiome research (e.g., sample handling and processing, data analysis and sharing), it is important to communicate best practices and lessons learned across the broader community. The user engagement session was a valuable opportunity for military user community representatives to provide information on specific operational needs for the tri-services and feedback on current efforts underway within this research community. Microbiome research remains a highly multi-disciplinary field and is an important component of biotechnology research within the DoD. Continued cooperation and coordination will facilitate effective leveraging of knowledge, skills and resources within this and the broader microbiome research community to address and provide solutions to DoD needs and interests.

As well as providing a forum to enhance collaboration, coordination, and communication of microbiome research among DoD organizations, the TSMC also engages academia and industry through annual Topical meetings. These focus on a specific topic within the microbiome field identified as a knowledge gap or emerging technology by the TSMC community. The TSMC Topical meetings invite leaders in those specific topics from academia and industry to present to the TSMC community to facilitate collaborations and assess new developments in the field.

## Electronic supplementary material

Below is the link to the electronic supplementary material.

Supplementary Material 1

## Data Availability

Not applicable.

## Declarations

### Disclaimer

All opinions or assertions contained herein are the private views of the authors and are not to be construed as official or reflecting the views of the Army, Navy, Air Force, or the Department of Defense. Any citations of commercial organizations and trade names in this report do not constitute an official Army, Navy, Air Force, or Department of the Defense endorsement or approval of the products or services of these organizations. Approved for public release; distribution is unlimited.

## Abbreviations

**AFRL**: US Air Force Research Laboratory
**BioMADE**: Bioindustrial Manufacturing and Design Ecosystem
**CBR**: Chemical and Biological Radiation
**CRREL**: Cold Regions Research and Engineering Laboratory
**COI**: Biotechnology Community of Interest
**DEVCOM SC**: US Army Combat Capabilities Development Command Soldier Center
**DoD**: Department of Defense
**ELSEI**: Ethical, Legal, Societal, and Environmental Implications
**EWS**: Enhanced Warfighter Systems
**GAA**: Guanidinoacetic acid
**GHG**: Greenhouse gas
**GI**: Gastrointestinal
**GTI**: Gastrointestinal tract infections
**JBADS**: Joint Biological Agent Decontamination System
**MIRECC**: Mental Illness Research, Education and Clinical Centers
**NMRC**: Naval Medical Research Center
**NMSC**: Navy Medical Service Corps
**NSMRL**: Naval Submarine Medical Research Laboratory
**ONR**: Office of Naval Research
**OWP**: Optimizing Warfighter Performance
**PACs**: Proanthocyanidins
**RR4**: Rodent research 4
**RTI**: Respiratory tract infections
**SBD**: Segmental bone defect
**SHIME**: Simulator of the Human Intestinal Microbial Ecology
**SMEs**: Subject matter experts
**SSTIs**: Skin and soft tissue infections
**TSMC**: Tri-Service Microbiome Consortium
**STO**: Science and Technology Operations
**USDA**: United States Department of Agriculture
**USAF**: United States Air Force
**USAFA**: United States Air Force Academy
**USARIEM**: US Army Research Institute of Environmental Medicine
**USNA**: United States Naval Academy
**USUHS**: Uniformed Services University of the Health Sciences
**WRAIR**: Walter Reed Army Institute of Research
