# Supplementary material for: Meeting report of the sixth annual tri-service microbiome consortium symposium
Source: Environ Microbiome. 2023 Aug 2;18:66. doi: 10.1186/s40793-023-00523-8 (PMC10399065; doi:10.1186/s40793-023-00523-8)
Supplement: Supplementary file 1 — Supplementary Material 1 [file 40793_2023_523_MOESM1_ESM.docx]

**Table S1**. Summary of presented research.

|  |  |  |
| --- | --- | --- |
| **Organization Type** | **Microbiome Area** | **Topic** |
| *DoD Service Laboratories* |  |  |
| *Air Force* |  |  |
| AFRL | Human | Changes in the composition of the gut microbiome during a prolonged submarine  deployment |
| AFRL | Enabler/*in vitro* | Gut-Muscle Axis Probiotic Evaluation Using *In vitro* Model Systems |
| AFRL | Environmental | The Effect of the Joint Biological Agent Decontamination System (JBADS) on  Aircraft-Associated Microbiology |
| AFRL | Environmental | Microbial Communities Vary by Function and Structure on Synthetic Polymers within  DoD Infrastructure |
| *Army* |  |  |
| DEVCOM SC | Enabler/*in vitro* | In vitro Skin Model for Enhanced Testing of Antimicrobial Textiles* |
| DEVCOM SC | Enabler/*in vitro* | Evaluation of Probiotic Growth Dynamics Using *In vitro* Batch fermentation* |
| DEVCOM SC | Enabler/*in vitro* | Using *in vitro* fermentation to model the human lower GI tract microbiome |
| EDRC-CRREL | Environmental | Whispers in the Dark: Sending Signal Waveforms Through Melanized Fungal Cultures |
| EDRC-CRREL | Environmental | Assessing microbial threats in thawing permafrost using metagenomic sequencing* |
| EDRC-CRREL | Environmental | Permafrost Thaw and the Carbon Cycle: Comparing Green House Gas Emissions from  Alaska and Abisko* |
| EDRC-CRREL | Environmental | Microbial Activity in Dust Contaminated Antarctic Snow* |
| EDRC-CRREL | Environmental | Microbial Activity in Arctic Soil: An Arctic Application of the DRTSPORE Mode* |
| EDRC-CRREL | Engineering/  environmental | Characterizing Microorganisms from Permafrost for Low-Temperature Synthetic Biology  Applications* |
| ERDC-CERL | Environmental | Investigating the Disturbed Soil Volatilome as a Novel Soil Sensing Tool* |
| USARIEM | Human | Orally Ingested Probiotics, Prebiotics, and Synbiotics as Countermeasures for Respiratory  and Gastrointestinal Tract Infections: A Systematic Review and Meta-analysis |
| WRAIR | Human/animal | Bioenergy Homeostasis: A Major Node in Functional Microbiome’s Stress Response Model |
| WRAIR | Human/animal | Delayed Impact of Radiation on Fecal Microbial Composition Depends on Gender |
| WRAIR | Enabler | Aerobiological Surveillance using NextGeneration Sequencing and Metagenomics  Analysis |
| WRAIR | Human | Impact of Testosterone Supplementation on the Fecal Microbiome and Metabolome  During Energy Deficit* |
| WRAIR | Human/animal | Spaceflight Induced Stress Caused Comprehensive Alteration of Fecal Microbiome  Beyond Bacteria* |
| WRAIR/MRSB | Human/animal | Pain Management During Space Mission In Context Of Gut-Brain Axis* |
| *Navy* |  |  |
| NSWC Indian Head | Enabler | Indian Head: Digital Engineering Biodefense |
| NRL | Environmental | Identifying Mycobiome from Aircraft Topcoat* |
|  |  |  |
| *DoD Academic Laboratories* |  |  |
|  |  |  |
| USAFA | Engineering/human | Fighting Pseudomonas aeruginosa Wound Infections with an Engineered Skin Microbe* |
| USAFA | Engineering/  environmental | Bioengineering and Optimization of Biocementation for Potential Space Applications* |
| USNA | Environmental | Metagenomic and Culture-based Characterization of the Chesapeake Bay Winter and  Summer Planktonic Microbiomes |
| USUHS | Human | Nasal microbiota evolution within the congregate setting imposed by military training |
| USUHS | Human/animal | Microbiome study in irradiated mice treated with BIO 300, a promising radiation  countermeasure |
| *Other Government Agency Laboratories* |  |  |
| DARPA | Engineering/  human | DARPA ReVector Program Aims to Reduce Mosquito Attraction by Engineering the  Human Skin Microbiome* |
| USDA | Human/animal | The effect of chlorinated drinking water on the gut microbiota: an *in vivo* analysis* |
| USDA-ARS | Enabler/*in vitro* | Insoluble rice bran fiber modifies gut microbial diversity in comparison to soluble fiber  in vitro* |
| USDA-ARS | Enabler/*in vitro* | Linking gut microbiome and metabolome shifts following probiotic administration |
| USDA-ARS | Human | Intestinal acylcarnitines and dysbiosis: implications for inflammatory bowel disease* |
| VA | Human | Medications and Gut, Oral, and Skin Microbiomes: Implications for Health and  Intervention: A United States-Veteran Microbiome Project (US-VMP) Study |
| VA | Enabler | Comparison of stabilized & non-stabilized gut microbiome samples via 16S rRNA  sequencing: A United States Veteran Microbiome Project (US-VMP) Study* |
| VA | Enabler | Intra- and Inter- Sequencing Center Variances for 16S rRNA using MiSeq Platform:  A United States-Veteran Microbiome Project (US-VMP) Study* |
|  |  |  |
| *Government-Affiliated Laboratories* |  |  |
| MIT | Human | Exploiting the battlefield microbiome^$^ |
| MIT | Human | Engineering microbes to improve human performance |
|  |  | |
| *Non-Government Industry Partners (including non-profit)* |  | |
| Draper | Enabler/*in vitro* | Immune Response Evaluation: A platform for evaluation of innate-adaptive immune  response to microbes and antigens* |
| BioMADE | Enabler | BioMADE, the Bioindustrial Manufacturing and Design Ecosystem* |

*Lightning talk; $Keynote; AFRL Air Force Research Laboratory; CERL Construction Engineering Research Laboratory; CRREL Cold Regions Research and Engineering Laboratory; DARPA Defense Advanced Research Projects Agency; DEVCOM Combat Capabilities Development Command; DoD US Department of Defense; EPA Environmental Protection Agency; ERDC US Army Engineer Research and Development Center; MRSB Medical Readiness Systems Biology; MIT Massachusetts Institute of Technology; NRL Naval Research Laboratory; NSWC Navy Surface Warfare Center (NSWC) Indian Head Division; SC Soldier Center; USAFA US Air Force Academy; USAREIM United States Army Research Institute of Environmental Medicine; USDA-ARS US Department of Agriculture – Agricultural Research Service; USNA US Naval Academy; USUHS Uniformed Services University of the Health Sciences; VA Department of Veterans Affairs; WRAIR Walter Reed Army Institute of Research
